# Myeloid-derived suppressor cell-derived osteoclasts with bone resorption capacity in the joints of arthritic SKG mice

**DOI:** 10.3389/fimmu.2024.1168323

**Published:** 2024-03-19

**Authors:** Yoshikazu Fujikawa, Sho Sendo, Alfonso del Peral Fanjul, Hirotaka Yamada, Kenichi Uto, Yuzuru Yamamoto, Takumi Nagamoto, Akio Morinobu, Jun Saegusa

**Affiliations:** ^1^Department of Rheumatology and Clinical Immunology, Kobe University Graduate School of Medicine, Kobe, Japan; ^2^Department of Clinical Laboratory, Kobe University Graduate School of Medicine, Kobe, Japan; ^3^Department of Rheumatology and Clinical Immunology, Kyoto University Graduate School of Medicine, Kyoto, Japan

**Keywords:** MDSCs, SKG mice, osteoclastogenesis, arthritis, microarray

## Abstract

**Background:**

Myeloid-derived suppressor cells (MDSCs) are heterogeneous immature myeloid cells with immunosuppressive functions. It is known that MDSCs are expanded at inflammatory sites after migrating from bone marrow (BM) or spleen (Sp). In chronic inflammatory diseases such as rheumatoid arthritis (RA), previous reports indicate that MDSCs are increased in BM and Sp, but detailed analysis of MDSCs in inflamed joints is very limited.

**Objective:**

The purpose of this study is to characterize the MDSCs in the joints of mice with autoimmune arthritis.

**Methods:**

We sorted CD11b^+^Gr1^+^ cells from joints (Jo), bone marrow (BM) and spleen (Sp) of SKG mice with zymosan (Zym)-induced arthritis and investigated differentially expressed genes (DEGs) by microarray analysis. Based on the identified DEGs, we assessed the suppressive function of CD11b^+^Gr1^+^ cells from each organ and their ability to differentiate into osteoclasts.

**Results:**

We identified MDSCs as CD11b^+^Gr1^+^ cells by flow cytometry and morphological analysis. Microarray analysis revealed that Jo-CD11b^+^Gr1^+^ cells had different characteristics compared with BM-CD11b^+^Gr1^+^ cells or Sp-CD11b^+^Gr1^+^ cells. Microarray and qPCR analysis showed that Jo-CD11b^+^Gr1^+^ cells strongly expressed immunosuppressive DEGs (*Pdl1, Arg1, Egr2* and *Egr3*). Jo-CD11b^+^Gr1^+^ cells significantly suppressed CD4^+^ T cell proliferation and differentiation *in vitro*, which confirmed Jo-CD11b^+^Gr1^+^ cells as MDSCs. Microarray analysis also revealed that Jo-MDSCs strongly expressed DEGs of the NF-κB non-canonical pathway (*Nfkb2* and *Relb*), which is relevant for osteoclast differentiation. In fact, Jo-MDSCs differentiated into osteoclasts *in vitro* and they had bone resorptive function. In addition, intra-articular injection of Jo-MDSCs promoted bone destruction.

**Conclusions:**

Jo-MDSCs possess a potential to differentiate into osteoclasts which promote bone resorption in inflamed joints, while they are immunosuppressive *in vitro*.

## 1 Introduction

Rheumatoid arthritis (RA) is a common autoimmune disease characterized by chronically inflamed joints. One of the current therapeutic goals for RA is to control disease activity in the early stages of inflammation to prevent the progression of joint destruction (1, 2). Many cytokines and chemokines are involved in the development of early RA and an imbalance between pro- and anti-inflammatory factors contributes to RA pathogenesis. Although pro- and anti- inflammatory mechanisms mediated by many cytokines and molecules have been investigated in earlier studies (3), anti-inflammatory mechanisms actually present in RA joints themselves have not been well investigated (4). Regulatory T cells (T-regs) and myeloid-derived suppressor cells (MDSCs) are two major cell groups with immunosuppressive functions (5). MDSCs in mice have been identified as a group of cells that co-express CD11b and Gr1 with suppressive function, but their phenotype is known to be diverse. Typical phenotype of mouse MDSCs is CD11b^+^Gr^+^CD11c^-^F4/80^+/-^CD124^+^ (6). MDSCs are heterogeneous cell populations of immature myeloid cells with immunosuppressive function (7). Precursors of MDSCs reside in the bone marrow (BM) in the steady state but migrate to inflamed organs under pathogenic conditions. In oncology, the role of MDSCs in tumor development has been well-investigated over the past several decades (8). MDSCs are induced at the affected sites by tumor cells and contribute to tumor growth by directly suppressing immune cells such as cytotoxic T cells via their production of cytokines including IL-10 and TGF-β, or indirectly by inducing T-regs. In rheumatology, unlike the role of T-regs, the function of MDSCs as suppressor cells is still controversial (9, 10). Previous reports concluded that MDSCs do play a crucial role in regulating mouse collagen-induced arthritis (CIA). Some reports indicated that MDSCs from the spleen of arthritic mice suppressed the proliferation of CD4^+^ T cells and their differentiation into Th17 cells *in vitro* (11, 12). On the other hand, another report concluded that splenic MDSCs are pro-inflammatory and aggravate arthritis in CIA (13). The CIA model is one of the most widely used mouse model of RA which is induced by immunization with type II collagen, however it has several limitations (14). First, the CIA model cannot show RA fluctuations and recurrences. Second, it has less extra-articular manifestations such as interstitial lung diseases, vasculitis or serositis, which is frequently involved in RA. In addition to induced arthritis models, the spontaneous arthritis models are useful for RA research. For example, K/BxN mice have a rapid onset of disease in a short period (15). On the other hand, SKG mice belong to T cell-mediated chronic autoimmune polyarthritis with slow progression (16). SKG mice has many characteristics which are similar to RA because 1) they initially start arthritis with interphalangeal joints of the forepaws, then progressing in a symmetrical fashion to swelling of other finger joints of the forepaws and hindpaws, and larger joints (wrists and ankles), 2) they develop arthritis chronically and progressively which results in bone and cartilage destruction, 3) they produce autoantibodies such as rheumatoid factor (RF), and 4) they develop extra articular manifestations like ILD. Therefore, we have used SKG mice as RA model and have already reported that MDSCs were increased in spleen and BM of arthritic SKG mice (17). We also reported that MDSCs in the inflamed lung had T cell suppressive function and could differentiate into tolerogenic dendritic cells during interstitial lung disease (18, 19). Most of previous reports analyzed MDSCs in spleen or BM (lymphoid organs), while little is known about MDSCs in inflamed joints. Only one report seems to have identified MDSCs in the joints of mice with proteoglycan-induced arthritis, a model of RA, in which granulocytic (G)-MDSCs obtained from the synovial fluid suppressed DC maturation (20). These findings indicate that MDSCs in inflamed tissues may have distinct functions across different organs. It is important to clarify the role of MDSCs in the local milieu of inflamed joints in order to understand pathophysiology such as the regulation of inflammation and the mechanism of bone destruction in the joints. Hence, the aim of the present study is to characterize MDSCs in the inflamed joints of arthritic SKG mice.

## 2 Materials and methods

### 2.1 Mice

Female SKG mice (CLEA JAPAN) were used at 4-12 weeks of age. All mice were kept under specific pathogen-free conditions at the Institute of Laboratory Animals, Graduate School of Medicine, Kobe University. Animal experiments were conducted in accordance with the protocol approved by the Animal Experimentation Committee of Kobe University.

### 2.2 Reagents and antibodies

Zym and 2-mercaptoethanol (2-ME) were purchased from Sigma-Aldrich, RPMI-1640 medium from Wako Pure Chemical Industries, fetal bovine serum (FBS) from MP Biomedicals, 1% penicillin/streptomycin from Lonza Walkersville, murine M-CSF and murine sRANKL from Pepro Tech. MEM α (Minimum Essential Medium α) was purchased from Gibco. Fluorescein isothiocyanate (FITC)-conjugated anti-Gr-1 (anti-Ly-6G/Ly-6C) (RB8-8C5), phycoerythrin (PE)-conjugated anti-Gr-1 (RB8-8C5), FITC-conjugated anti-rat IgG2b k isotype (MPC-11), PE-conjugated anti-mouse isotype (MPC-11), purified rat anti-mouse CD16/CD32 (Fc block) (2.4G2) and PerCP conjugated anti-mouse CD4 (RM4-5) were purchased from BD Biosciences. Peridinin chlorophyll protein (PerCP)-conjugated anti-CD11b (M1-70), Brefeldin A, allophycocyanin (APC)-conjugated anti-CD11b (M1-70), APC-conjugated anti-CD11c (N418), PE-conjugated anti-CD80 (16-10A1), PE-conjugated anti-CD274 (programmed death ligand 1 [PDL-1]) (MIH5), FITC-conjugated anti-CD4 (GK1.5), PerCP-conjugated anti-rat IgG2b k isotype (RTK4530), PE-Cy7 conjugated anti-mouse/rat FOXP3 (FJK-16s), PE conjugated anti-mouse/human T-bet (eBio4B10) and APC conjugated anti-mouse RORγt (B2D)were purchased from eBioscience. Cell staining buffer, FOXP3 Perm Buffer (10X), FOXP3 Fix/Perm Buffer (4X), FITC-conjugated anti-Ly-6G (1A8), APC-conjugated anti-Ly6C (HK1.4), FITC-conjugated anti-mouse CD182 (CXCR2) (SA044G4), FITC-conjugated anti-CD182 (CXCR2) (SA044G4), APC-conjugated anti-CD195 (CCR5) (HM-CCR5), APC-conjugated anti-mouse CX3CR1 (SA011F11), FITC-conjugated mouse IgG2b k isotype (MPC-11) and APC-conjugated mouse IgG2a k isotype (MOPC-173) were purchased from BioLegend. PE-Cy7 conjugated anti mouse NFκB2 p52 (polyclonal) and PE conjugated anti mouse Relb (polyclonal) were purchased from Bioss. The CFSE Cell Proliferation Kit for flow cytometry was purchased from Invitrogen.

### 2.3 Induction of arthritis in SKG mice

Each 8-week-old female SKG mouse (most of them are about 20 g/body) was intraperitoneally injected with 2 mg of Zym (Sigma-Aldrich) suspended in 0.5 mL normal saline as previously described (17).

### 2.4 Evaluation of arthritis

Severity of arthritis was assessed by scoring twice weekly as previously described (14). The maximum possible clinical arthritis score is 5.8, summed from the scores for the wrists, ankles and fingers of paws. Hind paws swelling was measured (mm) using a digital caliper (Mitutoyo QUICKmini, No.99MAN015M).

### 2.5 Isolation of tissues from BM, spleen and joints from SKG arthritic and non-arthritic mice

SKG mice were sacrificed with cervical dislocation after deep anesthesia. The body surface was disinfected with ethanol, an incision was made in the abdomen in a clean field and the spleen was extracted. Next, the femur was isolated and bone marrow cells were harvested. With the wrist and ankle joints in extension, an incision in the skin was made and the fatty tissue was carefully removed. Finally, the joint and swollen synovium are protectively harvested with scissors.

### 2.6 Sorting of CD11b^+^Gr1^+^ cells from BM, spleen and joints

Tissues were harvested as described above, then cells from each tissue were corrected passing through a cell strainer (70 μm), centrifuged (5min, 1500RPM), and erythrocytes were lysed by ACK. A uniform cell suspension was obtained pouring the sample through a cell strainer again and flow cytometry staining protocol was performed. BD FACSAria III (BD Biosciences) was used to sort CD11b^+^Gr1^+^ cells (MDSCs) from BM, spleen and joints of arthritic SKG mice (7-11 weeks after Zym injection). When sorting monocytic (M-) and granulocytic (G-) MDSCs, CD11b, Ly6G and Ly6C surface staining was performed.

### 2.7 Cell staining and flow cytometry

Single-cell suspensions from BM, spleen and joints were washed with cell staining buffer, preincubated with Fc block and stained with fluorescence-conjugated antibodies against CD11b, Gr1, Ly6G, Ly6C or CD11b, Gr1, CD80, F4/80, CD11c, MHC class II, and PDL1 for 30 minutes at 4°C for surface staining. For intranuclear staining, after surface markers staining is finished, the cell suspensions are fixed using FOXP3 Fixation/Permeabilization Buffer for 30 minutes at 4°C in darkness. Afterwards, the samples are washed twice with FOXP3 Permeabilization solution. The samples are incubated for 30 minutes with the intranuclear antibodies (Nfkb2, Relb, RORγt, T-bet or FOXP3) at room temperature while protected from light. After washing twice, the samples are resuspended in a suitable volume for flow cytometry. Flow cytometry data were acquired using a FACSVerse (BD Biosciences) and analyzed with FlowJo software (Tree Star).

### 2.8 May-Grunwald-Giemsa staining

CD11b^+^Gr1^+^ cells from BM, spleen and joints were stained with May-Grunwald-Giemsa to confirm their morphology after cytospin.

### 2.9 Microarray analysis

We commissioned Riken Genesis Co., Ltd. (Tokyo, Japan) to perform microarray analysis of isolated BM-CD11b^+^Gr1^+^ cells (1 x 10^6^ cells), Sp-CD11b^+^Gr1^+^ cells (1 x 10^6^ cells) and Jo-CD11b^+^Gr1^+^ cells (4.2 x 10^5^ cells). Briefly, total RNA of each sorted CD11b^+^Gr1^+^ cell population was extracted using Maxwell RSC simplyRNA Cells Kits (Promega) and its quality was estimated using NanoDrop ND-2000 (Thermo Fisher Scientific) and Agilent 4200 TapeStation (Agilent Technologies). cDNA was hybridized to GeneChip Mouse Genome 430 2.0 Array (Thermo Fisher Scientific) and was scanned at Riken Genesis Co. Raw data from microarrays were preprocessed and analyzed by Transcriptome Analysis Console (Thermo Fisher). We then performed, using transcriptome analysis console (TAC) software (Thermo Fisher Scientific), PCA analysis, heat map analysis and scatter plot analysis on the differentially expressed genes (DEGs) extracted by microarray analysis.

### 2.10 Quantitative real-time polymerase chain reaction

Total RNA was isolated from BM-CD11b^+^Gr1^+^ cells, Sp-CD11b^+^Gr1^+^ cells and Jo-CD11b^+^Gr1^+^ cells using RNeasy Mini Kits (Qiagen) followed by complementary DNA (cDNA) synthesis using a QuantiTect Reverse Transcription Kit (Qiagen). PCR mixtures were prepared using a QuantiTect SYBR Green PCR Kit (Qiagen) followed by quantitative PCR on a PikoReal system (Thermo Fisher Scientific). PCR primer pair sequences were as follows: glyceraldehyde-3-phosphate dehydrogenase (GAPDH), 5′-AACTTTGGCATTGTGGAAG-3′ (forward) and 5′-ACACATTGGGGGTAGGAACA-3′ (reverse); Arg1, 5’-CCAGAAGAATGGAAG-3’ (forward) and 5’-GCAGATATGCAGGGAGTCACC-3’ (reverse); iNOS, 5’-CACCAAGCTGAACTTGAGCG-3’ (forward) and 5’-CGTGGCTTTGGGCTCCTC-3’ (reverse); NOX2, 5’-TGTGGTTGGGGCTGAATGTC-3’ (forward) and 5’-CTGAGAAAGGAGAGCAGATTTCG-3’ (reverse); PD-L1, 5’-TGCTTCTCAATGTGACC-3’ (forward) and 5’-GGAACAACAGGATGGAT-3’ (reverse); Il-10, 5’-GGTTGCCAAGCCTTATCGGA-3’ (forward) and 5’-ACCTGCTCCACTGCCTTGCT-3’ (reverse); TGFβ, 5’-GCTAATGGTGGACCGCAACAAC-3’ (forward) and 5’-GCACTGCTTCCCGAATGTCTG-3’ (reverse); Nfkb2, 5’-CCAGCCCATCCATGACAGCA-3’ (forward) and 5’-GGAACACAATGGCATACTGTT-3’ (reverse); Relb, 5’-AGGATCTGCTTCCAGGCCTC-3’ (forward) and 5’-ATTCGGCAAATCCGCAGCTCT-3’ (reverse); Tnfrsf11a, 5’-CGAGGAAGATTCCCACAGAG-3’ (forward) and 5’-CAGTGAAGTCACAGCCCTCA-3’ (reverse); Traf6, 5’-GCCGAAATGGAAGCACAG-3’ (forward) and 5’-GGGCTATGGATGACAACAGG-3’ (reverse); Map3k14, 5’-GAGGCCGTGGAGAAGAGCC-3’ (forward) and 5’-GCATGGGCCACATTGTTGGG-3’ (reverse).

### 2.11 CD4^+^ T cell isolation

Splenocytes were isolated from 3-4 week-old SKG mice after erythrocyte lysis using ACK Lysing Buffer. CD4^+^ T cells were sorted from single cell suspensions of the splenocytes by biotinylated mAb against CD4, streptavidin-coated magnetic beads and a manual MACS system (Miltenyi Biotec) according to the manufacturer’s protocol.

### 2.12 T cell proliferation assay

CD4^+^ T cells were incubated with 10 μM CFSE according to the manufacturer’s protocol. The CD4^+^ T cells (1 x 10^5^/well) were cultured in RPMI supplemented with 10% FBS, 1% P/St and 50 µM 2-ME for 3 days in a 96-well flat-bottomed plate precoated with 10 µg/ml anti-CD3 antibody and 5 µg/ml anti-CD28 antibody. Isolated BM-CD11b^+^Gr1^+^ cells, Sp-CD11b^+^Gr1^+^ cells or Jo-CD11b^+^Gr1^+^ cells were co-cultured in direct contact with the CD4^+^ T cells and the proliferation of the CD4^+^ T cells was analyzed by measuring the CFSE fluorescence by flow cytometry.

### 2.13 T cell differentiation assay

Sorted CD4^+^ T cells (10^5^cells/well) and Jo-CD11b^+^ Gr1^+^ cells (10^5^cells/well) were co-cultured (direct contact) in CD3/CD28 precoated 96 well plates filled with 200µL per well of the medium for 5 days. Half of the culture medium was changed with fresh one every two days. The cells were collected from the bottom of the well and surface CD4 and intranuclear RORγt, T-bet and FOXP3 staining and subsequent flow cytometry analysis was performed.

### 2.14 Osteoclast differentiation

Isolated BM-CD11b^+^Gr1^+^ cells, Sp-CD11b^+^Gr1^+^ cells and Jo-CD11b^+^Gr1^+^ cells were stimulated with M-CSF (25 ng/ml) and RANKL (50 ng/ml) in 48-well plates at 1 x 10^5^ cells/well in 200 µL of MEM α supplemented with 10% FBS, 1% P/St on day 0. Half of the medium was changed every two days with fresh one and the cells were collected on day 10. Tartrate-resistant acid phosphatase-positive multinucleated cells (TRAP^+^ MNCs; ≥3 nuclei) were visualized using a TRAP staining kit (Cosmo Bio Co., Ltd) according to the manufacturer’s protocol and OCs were counted under a microscope (Keyence). Sorted M-MDSCs and G-MDSCs followed the same culturing process and were analyzed on day 7.

### 2.15 Bone resorption assay

A synthetic carbonate apatite coated 48 well plate (Bone resorption assay plate 48 well from Cosmo Bio Co., Ltd) was used to test the resorption capability of Jo-CD11b^+^Gr1^+^ cells. Using the above mentioned culturing method, Jo-CD11b^+^Gr1^+^ cells (5 x 10^4^ cells/well) were cultured in each well. On day 10, the medium was removed and each well was treated for 4 minutes with 5% sodium hypochlorite. After washing the plate with distilled water and letting it dry, microscopy images were acquired.

### 2.16 Intra-articular injection of Jo-MDSCs

High purity Jo-CD11b^+^Gr1^+^ cells from 3 arthritic SKG mice were collected and suspended in normal saline solution (NS). A total amount of 2.5x10^4^ cells/20µL of NS were injected in each left front and hind paws of 5 arthritic SKG mice. On the opposite side, 20µL of NS was injected as a control. After 3 weeks the mice were sacrificed and tissues properly stored and analyzed.

### 2.17 Histological and radiological assessment of mouse joints

Hind paws were collected and immediately placed in 4% paraformaldehyde phosphate buffer solution (Wako Pure Chemical, Osaka, Japan). Ultrahigh-resolution micro CT images were acquired using an R_mCT2 (Rigaku, Tokyo, Japan) (tube voltage 90 kV, current 160 μA, exposure time 3 min). After the micro CT images were obtained, a decalcification process of the joints with 10% EDTA/2Na solution (MUTO PURE CHEMICALS, Tokyo, Japan) was performed for one week, changing the medium twice. Hematoxylin-eosin (H&E) and unstained histological slides were commissioned to Pathotech Ltd. TRAP staining was performed after deparaffinization and rehydration of the unstained slides with serial dilutions of xylene and ethanol, following 2 hours of incubation with the TRAP staining solution (TRAP staining kit, Cosmo Bio Co., Ltd). High resolution images of H&E and TRAP staining were obtained with a Keyence All-in-O Fluorescence Microscope BZ-X800.

### 2.18 Statistical analysis

Results are expressed as the mean ± SEM. All analyses were performed using GraphPad Prism 9 software. Statistical comparisons between 2 groups were performed using paired t test. For multiple comparisons, we used ordinary or RM one-way ANOVA followed by Tukey’s multiple comparisons test or Kruskal-Wallis test followed by Dunn’s multiple comparisons test according to the distribution normality of the data. P values ≤0.05 were considered significant.

## 3 Results

### 3.1 CD11b^+^Gr1^+^ cells accumulate in the joints of arthritic SKG mice

**Figure 1:**
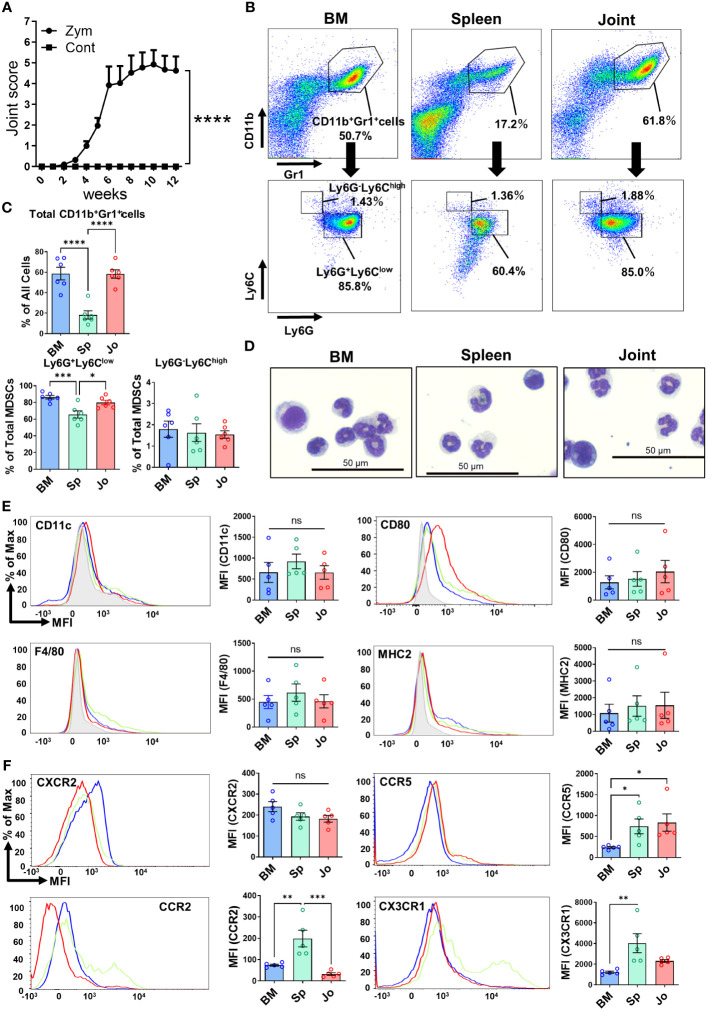
Detection of BM-, Sp- and Jo-CD11b^+^Gr^+^ cells from SKG mice with autoimmune arthritis. **(A)** Progression of arthritis in Zym-injected SKG mice compared with normal saline-injected SKG mice (Control). **(B)** Cells were isolated from BM, spleen or joints of arthritic mice at 7-11 weeks and analyzed by flow cytometry. Upper; Representative plots of total CD11b^+^Gr1^+^ cells, Lower; Representative plots of G-CD11b^+^Gr^+^ cells (CD11b^+^Ly6G^+^Ly6C^low^) and M-CD11b^+^Gr^+^ cells (CD11b^+^Ly6G^-^Ly6C^high^). **(C)** Comparison of total CD11b^+^Gr^+^ cells, G-CD11b^+^Gr^+^ cells and M-CD11b^+^Gr^+^ cells from each tissue (n=6 in each). **(D)** May-Giemsa staining of CD11b^+^Gr1^+^ cells isolated from BM, spleen and joints of arthritic SKG mice. **(E)** Representative plots of surface markers on CD11b^+^Gr^+^ cells from BM (n=5), spleen (n=5) and joints (n=5). **(F)** Representative plots of chemokine receptors on CD11b^+^Gr^+^ cells (n=5 in each). The filled gray histograms in **(E, F)** shows the isotype controls. Each point represents a sample from an individual mouse. **p≤0.01, ****p ≤0.0001, ***p ≤0.001, **p≤0.01, *p ≤0.05, ns; no significant by ordinary one-way ANOVA followed by Tukey’s multiple comparisons test.

It is known that the injection of Zym induces chronic arthritis in SKG mice. The mechanism relies on a spontaneous point mutation of ZAP70 in this mouse strain (16, 21). In our experiments, SKG mice developed arthritis 6 weeks after Zym injection and inflammation remained present for up to 12 weeks (**Figure 1A**). We isolated CD11b^+^Gr1^+^ cells from the joints of arthritic SKG mice and non-arthritic mice. While the proportion of Jo-CD11b^+^Gr1^+^ cells is around 60% in arthritic mice (**Figure 1B**), only a few (<1%) were present in non-arthritic mice (**Supplementary Figure S1**). We then determined the characteristics of CD11b^+^Gr1^+^ cells in inflamed joints, bone marrow and spleen of these mice with chronic arthritis. Flow cytometric analysis revealed that the proportion of CD11b^+^Gr1^+^ cells was different in the three organs (**Figures 1B**; upper, **1C**; upper). The proportion of CD11b^+^Gr1^+^ cells was higher in joints and BM than spleen. We further phenotyped subpopulations of CD11b^+^Gr1^+^ cells and characterized CD11b^+^Ly6G^+^Ly6C^low^ cells and CD11b^+^Ly6G-Ly6C^high^ cells which mark G-MDSCs and M-MDSCs respectively (6, 7). The proportion of CD11b^+^Ly6G^+^Ly6C^low^ cells was higher than CD11b^+^Ly6G-Ly6C^high^ cells in each organ (**Figures 1B**; lower, **1C**; lower), and the proportion of CD11b^+^Ly6G^+^Ly6C^low^ cells in joints and BM was significantly higher than spleen.

We next isolated CD11b^+^Gr1^+^ cells from the three organs and compared their morphology by May-Grunwald-Giemsa (MGG) staining (**Figure 1D**). CD11b^+^Gr1^+^ cells consisted of two morphologically different cell types, one with pseudo-segmented or ring-shaped nuclei reminiscent of granulocyte (G-CD11b^+^Gr1^+^ cells) and the other mononuclear monocyte-like cells (M-CD11b^+^Gr1^+^ cells). The proportion of G-CD11b^+^Gr1^+^ cells was higher than the M-CD11b^+^Gr1^+^ cells. These G-CD11b^+^Gr1^+^ cells were similar to neutrophils but did not have any granules, suggesting that they were not in fact neutrophils. Overall, there were no clear morphological differences among the CD11b^+^Gr1^+^ cells in each organ.

We then sought phenotypic differences of the CD11b^+^Gr1^+^ cells in the three organs (**Figure 1E**). It is known that MDSCs usually lack the surface markers of differentiated myeloid cells such as CD11c, F4/80 and MHC class II. We found that none of the CD11b^+^Gr1^+^ cells in each organ expressed these markers (7, 22). CD11b^+^Gr1^+^ cells in joints expressed slightly more CD80 than those in BM or spleen, but this difference did not reach statistical significance, consistent with previous reports (23).

Immature myeloid cells (IMCs) are generated from hematopoietic stem and progenitor cells (HSPCs) in the BM. HSPCs also migrate from the BM to spleen and secondary lymph nodes (LNs). Under conditions of chronic inflammation, maturation of IMCs into fully differentiated cells is inhibited, while they retain their suppressive activity, which results in MDSC generation (6). Chemokines are associated with chemo-attraction of myeloid cells, either from the BM to the blood (mobilization); from the blood to sites of inflammation (recruitment); or from tissues and blood to the LNs (24). Mobilization and recruitment of myeloid cells is directed by specific chemokine receptors on the cell surface (25). Thus, we assessed the expression of chemokine receptors on CD11b^+^Gr1^+^ cells from each organ. We found that Jo-CD11b^+^Gr1^+^ cells expressed more CCR5 than BM-CD11b^+^Gr1^+^ cells, whereas Sp-CD11b^+^Gr1^+^ cells expressed more CCR2, CCR5 and CX3CR1 than BM-CD11b^+^Gr1^+^ cells. In contrast, CXCR2 expression did not differ among the CD11b^+^Gr1^+^ cells from each organ (**Figure 1F**). These results suggest that CD11b^+^Gr1^+^ cells from each organ have different chemotactic potential.

### 3.2 Jo-CD11b^+^Gr1^+^ cells have differential gene expression compared with BM- or Sp-CD11b^+^Gr1^+^ cells

**Figure 2:**
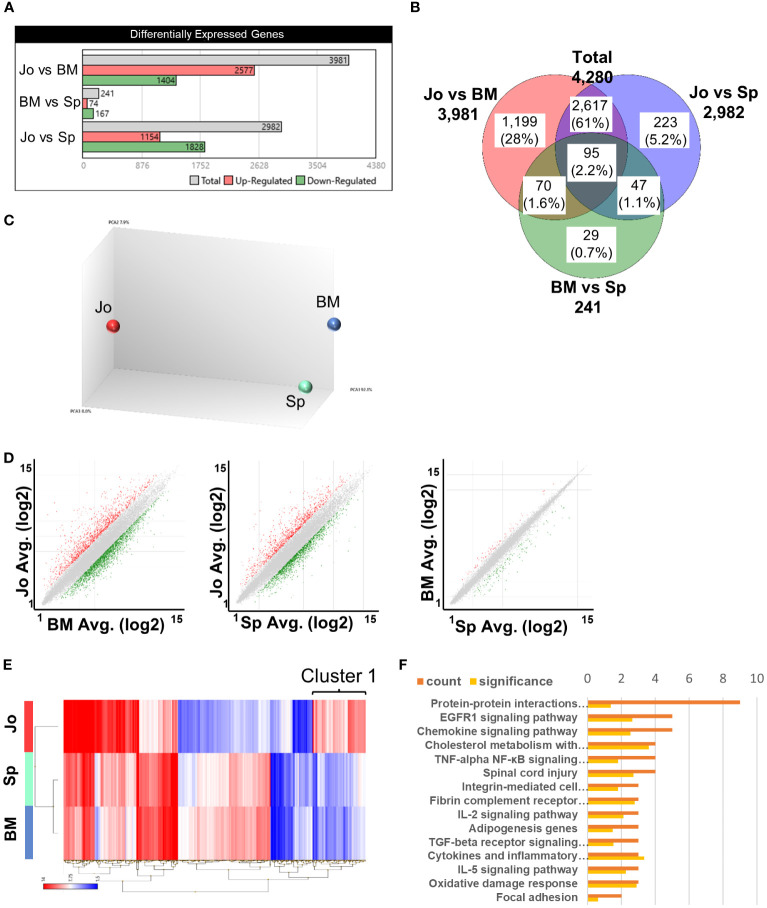
Microarray analysis of CD11b^+^Gr^+^ cells from BM, spleen and joints. **(A)**, Differentially expressed genes (DEGs) in CD11b^+^Gr^+^ cells from 3 tissues. **(B)** Venn diagram of DEGs in BM-CD11b+Gr+ cells, Sp-CD11b+Gr+ cells and Jo-CD11b+Gr+ cells; total DEGs = 4280 genes. **(C)** Principal components analysis (PCA) of BM-, Sp- and Jo-CD11b+Gr+ cells. **(D)** Scatter plot analysis comparing between Jo- vs BM-MDSCs, Jo- vs Sp-MDSCs or BM- vs Sp-MDSCs; fold change ≤ -3 and ≥ 3. **(E)** Heat map of microarray data showing hierarchical clustering of 663 DEGs in MDSCs from BM, spleen and joints. Red or blue colors indicate up- or downregulated genes respectively; fold change ≤ -3 (blue) and ≥ 3 (red). **(F)** Pathway enrichment analysis of cluster 1 on E (cluster 1 in which DEGs of Jo-CD11b+Gr1+ cells were upregulated and those of BM- and Sp-CD11b+Gr1+ cells were downregulated). The orange bar in the upper row is the number of genes and the yellow bar in the lower row is the significant difference.

To characterize Jo-CD11b^+^Gr1^+^ cells in inflamed joints, we next sorted CD11b^+^Gr1^+^ cells from BM, spleen and joints of arthritic SKG mice (**Supplementary Figure S2**) and performed microarray analysis. The total number of differentially-expressed genes (DEGs) among CD11b^+^Gr1^+^ cells in the three organs was 4,280 (**Figures 2A, B**). There were 3,981 and 2982 DEGs distinguishing Jo-CD11b^+^Gr1^+^ cells from BM-CD11b^+^Gr1^+^ cells and from Sp-CD11b^+^Gr1^+^ cells, respectively. On the other hand, there were only 241 DEGs that distinguished BM-CD11b^+^Gr1^+^ cells from Sp-CD11b^+^Gr1^+^ cells. Principal component analysis (PCA) showed that Jo-CD11b^+^Gr1^+^ cells formed populations separate from both BM- and Sp-CD11b^+^Gr1^+^ cells (**Figure 2C**). Scatter plot analysis also indicated that there were many DEGs between Jo-CD11b^+^Gr1^+^ cells and BM-/Sp-CD11b^+^Gr1^+^ cells, while relatively few DEGs were found between BM-CD11b^+^Gr1^+^ cells and Sp-CD11b^+^Gr1^+^ cells (**Figure 2D**). In the scatter plots described above, a total of 663 DEGs exhibited fold-changes ≧3 (red) or ≦-3 (green). We generated a heat map of CD11b^+^Gr1^+^ cells from the three organs using these 663 genes (**Figure 2E**). DEGs of Jo-CD11b^+^Gr1^+^ cells seemed to be different from those of BM-/Sp-CD11b^+^Gr1^+^ cells, while DEGs of BM-CD11b^+^Gr1^+^ cells and Sp-CD11b^+^Gr1^+^ cells were relatively similar to each other. To characterize Jo-CD11b^+^Gr1^+^ cells, we focused on cluster 1 in which DEGs of Jo-CD11b^+^Gr1^+^ cells were upregulated relative to BM-/Sp-CD11b^+^Gr1^+^ cells (**Figure 2E**). There were 117 DEGs in cluster 1, for which we performed pathway enrichment analysis (**Figure 2F**). This revealed that cluster 1 contained many genes associated with inflammation-related pathways including pro- and anti- inflammatory genes. This cluster also contained TNF-α and NF-κB-related pathway genes which included canonical and non-canonical pathways related to osteoclast differentiation.

These results suggested that Jo-CD11b^+^Gr1^+^ cells were distinct from BM-/Sp-CD11b^+^Gr1^+^ cells, and that they potentially had pro- or anti-inflammatory and osteoclast differentiation abilities.

### 3.3 Jo-CD11b^+^Gr1^+^ cell properties are consistent with MDSCs and they suppress T cell-proliferation and differentiation *in vitro*

**Figure 3:**
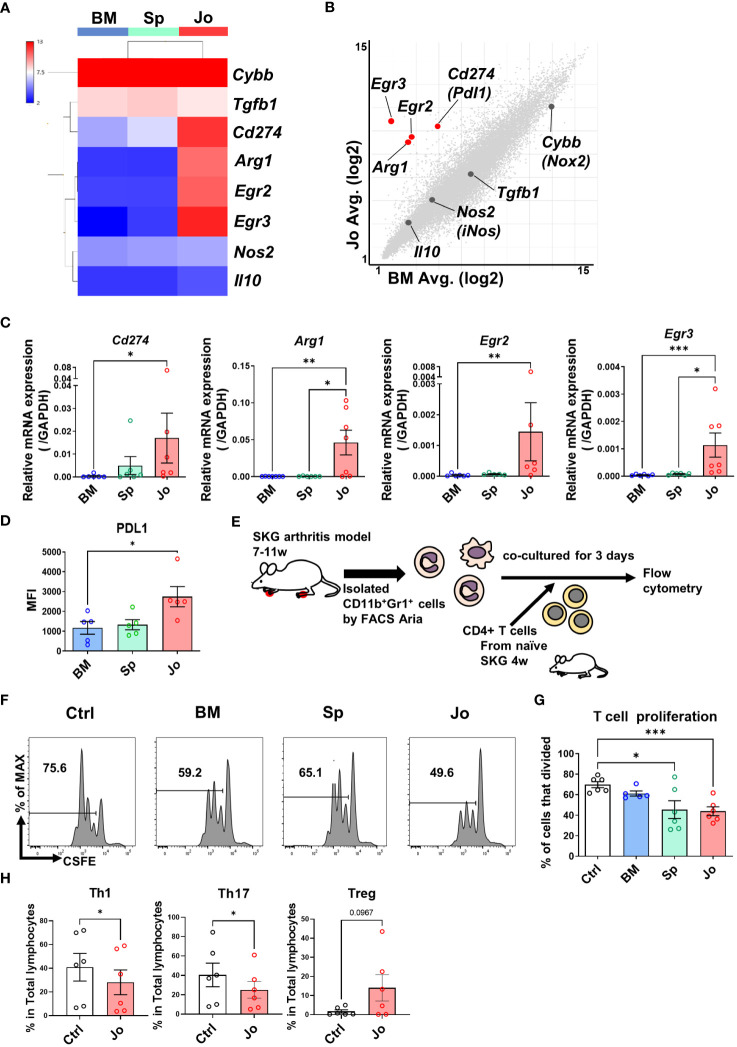
Jo-CD11b^+^Gr^+^ cells and Sp-CD11b^+^Gr^+^ cells suppress CD4^+^ T cell proliferation. **(A)** Heat map of the immunosuppressive genes (*Cybb* (*Nox2*), *Tgfb1*, *Cd274* (*Pdl1*), *Arg1*, *Egr2*, *Egr3*, *Nos2* (*iNos*) and *Il10*) in BM-/Sp-/Jo-CD11b^+^Gr^+^ cells. **(B)** Scatter plot of suppressive genes in Jo- vs BM-CD11b^+^Gr^+^ cells (Red dots; fold change ≤ -4 and **≥** 4, X and Y axes are logarithmic). **(C)** mRNA of DEGs (*Pdl1*, *Arg1*, *Egr2* and *Egr3*) in BM-/Sp-/Jo-CD11b^+^Gr^+^ cells quantified by qPCR (n=7 each). **(D)** Flow cytometry analysis of PDL1 in BM-/Sp-/Jo-CD11b^+^Gr^+^ cells. **(E)** Schematic experimental method for panel **(F, G)**. Isolated CD4^+^ T cells were labeled with CFSE, stimulated with CD3 and CD28, co-cultured with or without BM-/Sp-/Jo-CD11b^+^Gr^+^ cells for 3 days and then analyzed by flow cytometry. **(F)** Representative flow cytometry images of CD4^+^ T cell proliferation co-cultured with or without BM-/Sp-/Jo-CD11b^+^Gr^+^ cells; histogram of CFSE-labeled CD4^+^ T cell proliferation. **(G)** Quantitative analysis of proliferated T cells in each group (each point on boxplot shows the frequency of proliferating T cells; n=6 each). **(H)** T cell differentiation assay: proportion of Th1, Th17 and Treg cells co-cultured with or without Jo-CD11b^+^Gr^+^ cells (n=6). Data are shown as mean ± SEM. ***p≤0.001, **P ≤0.01, *P ≤0.05, by Kruskal-Wallis test followed by Dunn’s multiple comparisons test **(C)**, by one-way ANOVA followed by Tukey’s multiple comparisons test **(D, G)** and by Paired t test **(H)**.

Some previous reports have confirmed that splenic cells with an MDSC phenotype had immunosuppressive functions in arthritis models (10, 26). However, little is known about the phenotype and function of MDSCs in the inflamed joints. Therefore, we investigated the suppressive properties of Jo-CD11b^+^Gr1^+^ cells. We first analyzed genes associated with immunosuppressive functions (*Cybb, Tgfb1, Cd274 (PDL1), Arg1, Egr2, Egr3, Nos2* and *Il10*) among the CD11b^+^Gr1^+^ cells in each organ using heat-mapped microarray data. This revealed that *Cd274, Arg1, Egr2* and *Egr3* were more highly expressed in Jo-CD11b^+^Gr1^+^ cells than BM-/Sp-CD11b^+^Gr1^+^ cells (**Figure 3A**). Scatter plot analysis comparing between Jo-CD11b^+^Gr1^+^ cells and BM-CD11b^+^Gr1^+^ cells or between Jo-CD11b^+^Gr1^+^ cells and Sp-CD11b^+^Gr1^+^ cells also showed that *Cd274, Arg1, Egr2* and *Egr3* were highly expressed in the former (**Figure 3B**; **Supplementary Figure S3B**). We next quantified the expression of these suppressive genes (*Cybb*, *Tgfb1, Cd274, Arg1, Egr2, Egr3, Nos2* and *Il10*) in CD11b^+^Gr1^+^ cells from each organ using quantitative real-time PCR. Jo-CD11b^+^Gr1^+^ cells exhibited significantly greater expression of *Cd274*, *Arg1*, *Egr2* and *Egr3* than BM-CD11b^+^Gr1^+^ cells (**Figure 3C**). Furthermore, the levels of *Arg1* and *Egr3* in Jo-CD11b^+^Gr1^+^ cells were significantly higher than in Sp-CD11b^+^Gr1^+^ cells (**Figure 3C**), whereas *Cybb*, *Tgfb1*, *Nos2* and *Il10* were not different among them (**Supplementary Figure S3A**). Flow cytometric analysis confirmed that PDL1 was significantly higher in Jo-CD11b^+^Gr1^+^ cells than BM-CD11b^+^Gr1^+^ cells (**Figure 3D**). These results suggest that Jo-CD11b^+^Gr1^+^ cells might exert strong immunosuppression. To confirm their suppressive function, we co-cultured CD4^+^ T cells with or without BM-, Sp- or Jo-CD11b^+^Gr1^+^ cells (**Figure 3E**). Jo-CD11b^+^Gr1^+^ cells, and also Sp-CD11b^+^Gr1^+^ cells, significantly suppressed T cell proliferation (44 ± 4% and 70 ± 3% proliferating T cells in the presence of Jo-MDSCs and control cells, respectively; p<0.01) (**Figures 3F, G**). Furthermore, RORγt^+^ (Th17) and T-bet^+^ (Th1) CD4^+^ T cell populations were significantly decreased if co-cultured with Jo- CD11b^+^Gr1^+^ cells. This indicates that Jo-CD11b^+^Gr1^+^ cells can inhibit the differentiation of naïve T cells into effector Th1 and Th17 T cells. While Jo- CD11b^+^Gr1^+^ cells tended to increase the Treg population, although this result did not reach statistical significance (**Figure 3H**). Taken altogether, the above results indicated that the properties of Jo-CD11b^+^Gr1^+^ cells are consistent with MDSCs.

### 3.4 Jo-MDSCs express genes associated with osteoclastogenesis and can differentiate into functional osteoclasts *in vitro*

**Figure 4:**
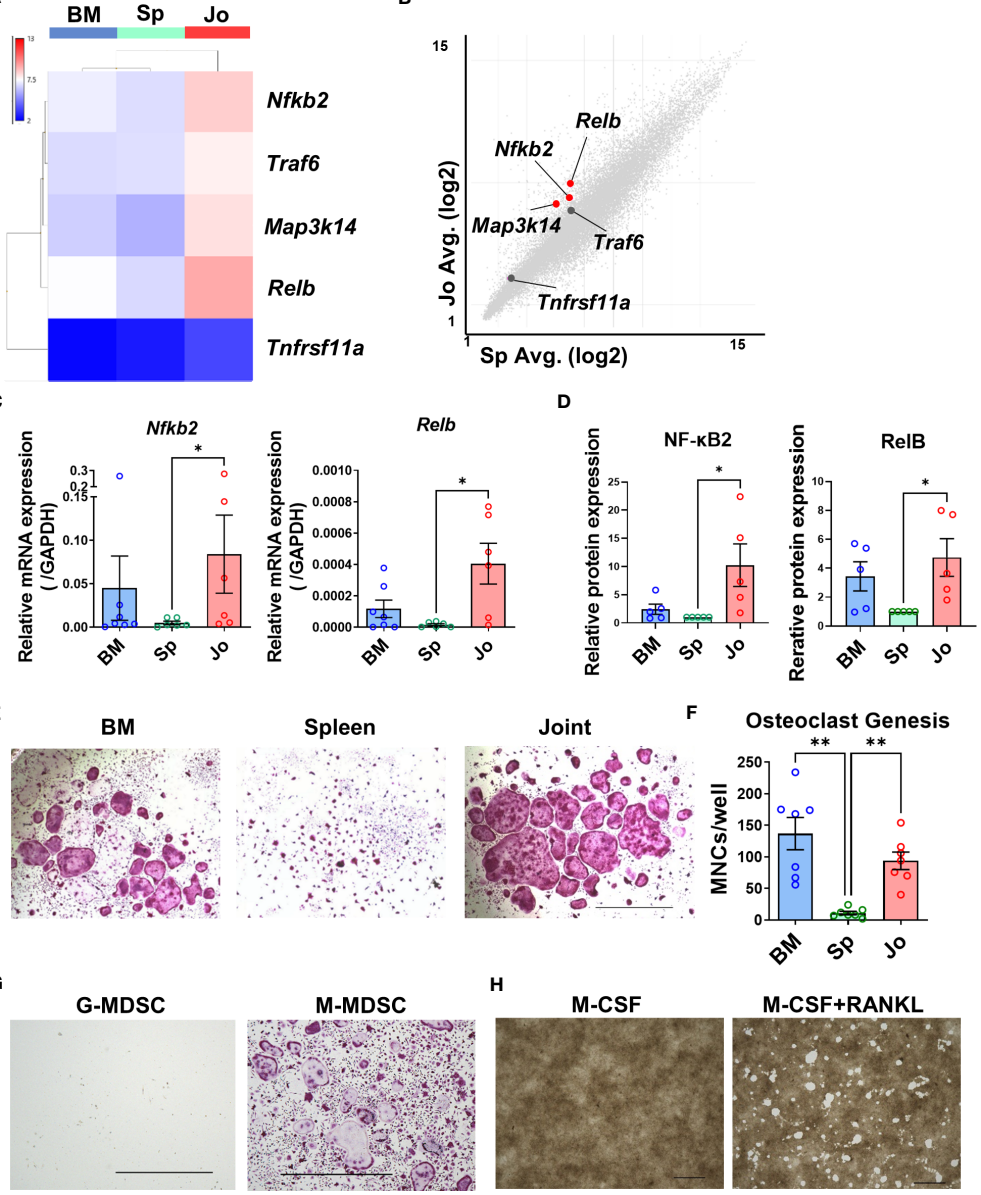
Jo-MDSCs possess functional osteoclast differentiation potential. **(A)** Heat map of non-canonical NF-κB pathway genes (*Nfkb2*, *Traf6*, *Map3k14*, *Relb* and *Tnfrsf11a*). **(B)** Scatter plot analysis of non-canonical NF-κB pathway genes in Jo-MDSCs and Sp-MDSCs (Red dots; fold change **≤** -4 and **≥** 4, X and Y axes are logarithmic). **(C)** Relative mRNA expression of *Nfkb2* and *Relb* in BM-CD11b^+^Gr^+^ cells, Sp-/Jo-MDSCs quantified by qPCR (n=7 each). **(D)** Relative protein expression of NF-κβ2 and RelB in BM-CD11b^+^Gr^+^ cells, Sp-/Jo-MDSCs by Flow cytometry (n=5 each). **(E)** Representative images of TRAP staining-positive cells differentiated from BM-CD11b^+^Gr^+^ cells, Sp-/Jo-MDSCs stimulated by M-CSF and RANKL for 10 days (n=7 each). Scale bar 1000μm. **(F)** Count of multi-nucleated cells (MNCs)/well in each group (each point on boxplot shows the counts of MNCs per well; n=7 each). **(G)** Representative images of TRAP staining-positive cells differentiated from sorted G-MDSCs or M-MDSCs of inflamed joints, stimulated with M-CSF and RANKL for 7 days (Performed 3 independent experiments). Scale bar 1000μm. **(H)** Resorption of synthetic carbonate apatite coated well. Jo-MDSCs were stimulated with M-CSF or with M-CSF & RANKL, respectively (48 well plate, 10 days culture. Performed 3 independent experiments). Scale bar 1000μm. Data are shown as mean ± SEM. **P ≤0.01, *P ≤0.05, by Kruskal-Wallis test followed by Dunn’s multiple comparisons test **(C)** and by RM one-way ANOVA followed by Tukey’s multiple comparisons test **(F)**.

Some previous reports have indicated that MDSCs can differentiate into osteoclasts in other models (4, 27). As we showed in the pathway enrichment analysis, Jo-MDSCs had upregulated genes related to the NF-κB pathway (**Figure 2F**). It is known that NF-κB canonical and non-canonical pathways are both associated with osteoclast differentiation (28, 29). Our microarray analysis revealed that Jo-MDSCs more strongly expressed genes associated with the NF-κB non-canonical than the canonical pathway (**Figure 4B**; **Supplementary Figure S4**). The heat map of NF-κB non-canonical pathway genes revealed that *Nfkb2*, *Traf6*, *Map3k14* and *Relb* were highly expressed in Jo-MDSCs compared with BM-CD11b^+^Gr1^+^ cells or Sp-MDSCs (**Figure 4A**). Scatter plot analysis comparing Jo-MDSCs and Sp-MDSCs also showed that *Nfkb2*, *Map3k14* and *Relb* were highly expressed in Jo-MDSCs (**Figure 4B**). On the other hand, expression levels of these genes are relatively similar between Jo-MDSCs and BM-CD11b+Gr1+ cells or between BM-CD11b+Gr1+ cells and Sp-MDSCs (**Supplementary Figure S5B**). We next quantified the expression of these NF-κB non-canonical pathway genes (*Nfkb2*, *Traf6*, *Map3k14*, *Relb* and *Tnfrsf11a*) using qPCR (**Figure 4C**; **Supplementary Figure S5A**), showing that *Nfkb2* and *Relb* were more highly expressed in Jo-MDSCs than Sp-MDSCs. This result was verified at the protein levels using flow cytometry, showing a significant increase in the relative expression of NF-κB2 and RelB levels in Jo-MDSCs compared with those in Sp-MDSCs (**Figure 4D**) These results also suggested that Jo-MDSCs may have osteoclast differentiation potential. Therefore, we stimulated isolated BM-CD11b^+^Gr1^+^ cells, Sp-MDSCs and Jo-MDSCs with M-CSF and RANKL *in vitro* to directly determine their osteoclast differentiation ability. TRAP staining showed that Jo-MDSCs and BM-CD11b^+^Gr1^+^ cells but not Sp-MDSCs could differentiate to osteoclasts (**Figures 4E, F**).

Within the two populations (G-MDSCs and M-MDSCs) that have been described, we sought to elucidate which population in the joints do have the differentiation potential towards osteoclasts. After sorting both populations and culturing them with M-CSF and RANKL for 7 days, we proceeded with the TRAP staining. We discovered that the ability to develop into osteoclasts was limited to M-MDSCs (**Figure 4G**). M-MDSCs had a fibroblast-like phenotype when cultured and quickly adhered to the well’s bottom. They swiftly started to proliferate and eventually merged together becoming osteoclasts. On the other hand, G-MDSCs barely attached to the bottom and most of them died within a few days of culture.

We further examined whether the osteoclasts differentiated from Jo-MDSCs actually have a bone resorptive function, and revealed that Jo-MDSC-OCs do had a potential to resorb synthetic carbonate apatite *in vitro* (**Figure 4H**).

### 3.5 Osteoclastic Jo-MDSCs accelerated bone resorption *in vivo*

**Figure 5:**
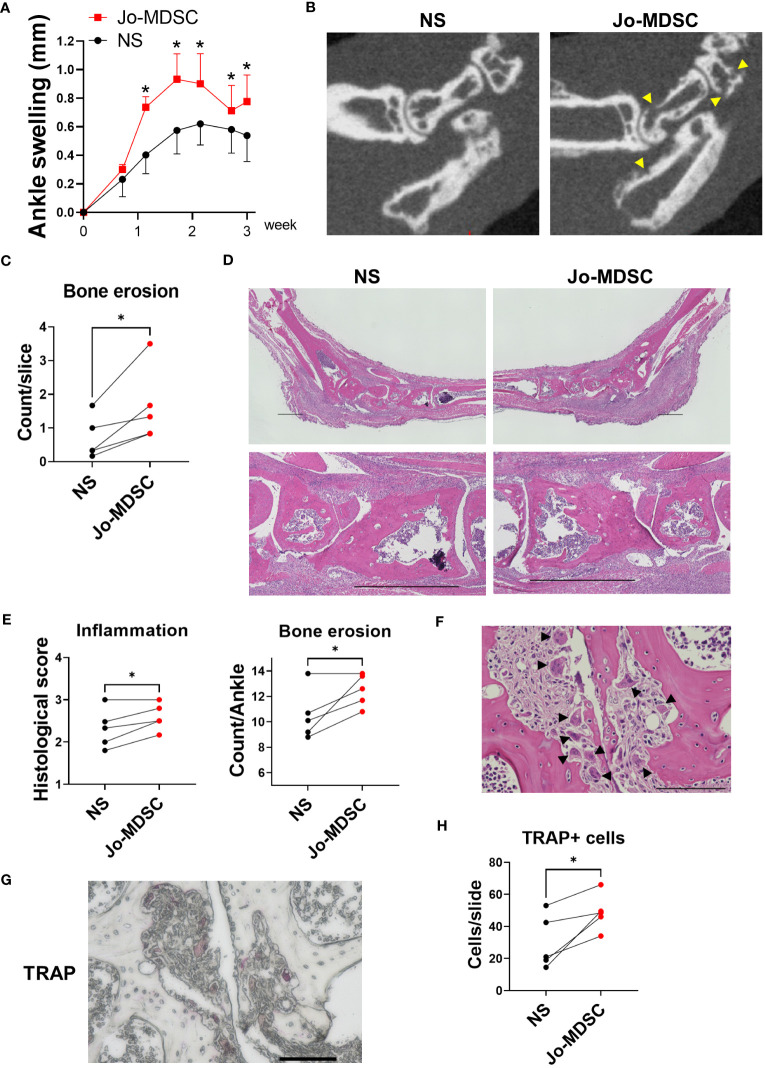
Intra-articular injection of Jo-MDSCs aggravate joint swelling and bone destruction *in vivo*. **(A)** Clinical course of ankle swelling (mm) of five arthritic SKG mice which received intra-articular injection of Jo-MDSCs (left hind paw) and NS (right hind paw). **(B)** Representative micro CT scans of hind paws from the intra-articular injected SKG mice. Arrowhead shows bone erosions. **(C)** Mean bone erosion count from micro CT scans assessed by two independent observers. **(D)** Representative H&E staining images of ankle joints of intra-articular injected SKG mice and its control. Scale bar 1000μm. **(E)** Synovial inflammation and bone erosion semi-quantitative histological scoring of H&E specimens from intra-articular injected SKG mice (n=5). **(F)** Representative H&E staining image of osteoclasts around bone resorptive area of Jo-MDSC injected joint. Arrowhead shows osteoclasts. Scale bar 100μm. **(G)** TRAP staining; representative TRAP positive cells in bone resorptive areas. Scale bar 100μm. **(H)** Quantitative evaluation of TRAP positive cells; mean count per slide assessed by two independent observers. Data are shown as mean ± SEM in Panel **A**. *P ≤0.05, ns; no significant by Paired t test.

We examined whether Jo-MDSCs, which had osteoclastogenic potential *in vitro*, had the same function as osteoclasts in inflammatory joints. To validate the function of Jo-MDSCs *in vivo*, we performed intra-articular injection of Jo-MDSCs into lateral joints of arthritic SKG mice. The ankle swelling was worsened in the Jo-MDSCs injected side (**Figure 5A**). To assess the degree of bone damage, we followed by performing a microCT. The Jo-MDSCs injected side had a statistical significant increase in bone erosions (**Figures 5B, C**). Next, we evaluated the H&E stained joint slides and obtained a semi-quantitative scores of synovial inflammation and bone erosion, according to the “SMASH” recommendations for microscopic histological scoring of arthritis animal models (30). The Jo-MDSCs injected side showed a significant increase in the severity of synovial inflammation and bone erosions (**Figures 5D, E**). We identified remarkable numbers of osteoclasts close to bone resorptive area in the Jo-MDSCs injected joints (**Figure 5F**). Lastly, we performed TRAP staining and counted the number of osteoclasts in the cortical of the hind paws’ major bones (tibia, talus, calcaneus, navicular, cuneiform and metatarsal bones). A significant increase in the number of osteoclasts was obtained in the Jo-MDSCs injected side (**Figures 5G, H**).

Based on these results, we showed that Jo-MDSCs indeed aggravate arthritis, and mainly increased bone resorption, in the joints from SKG mice. It is most likely that the injected Jo-MDSCs developed into osteoclasts amidst the inflammatory milieu within the joint and in turn exacerbated bone destruction.

**Figure 6:**
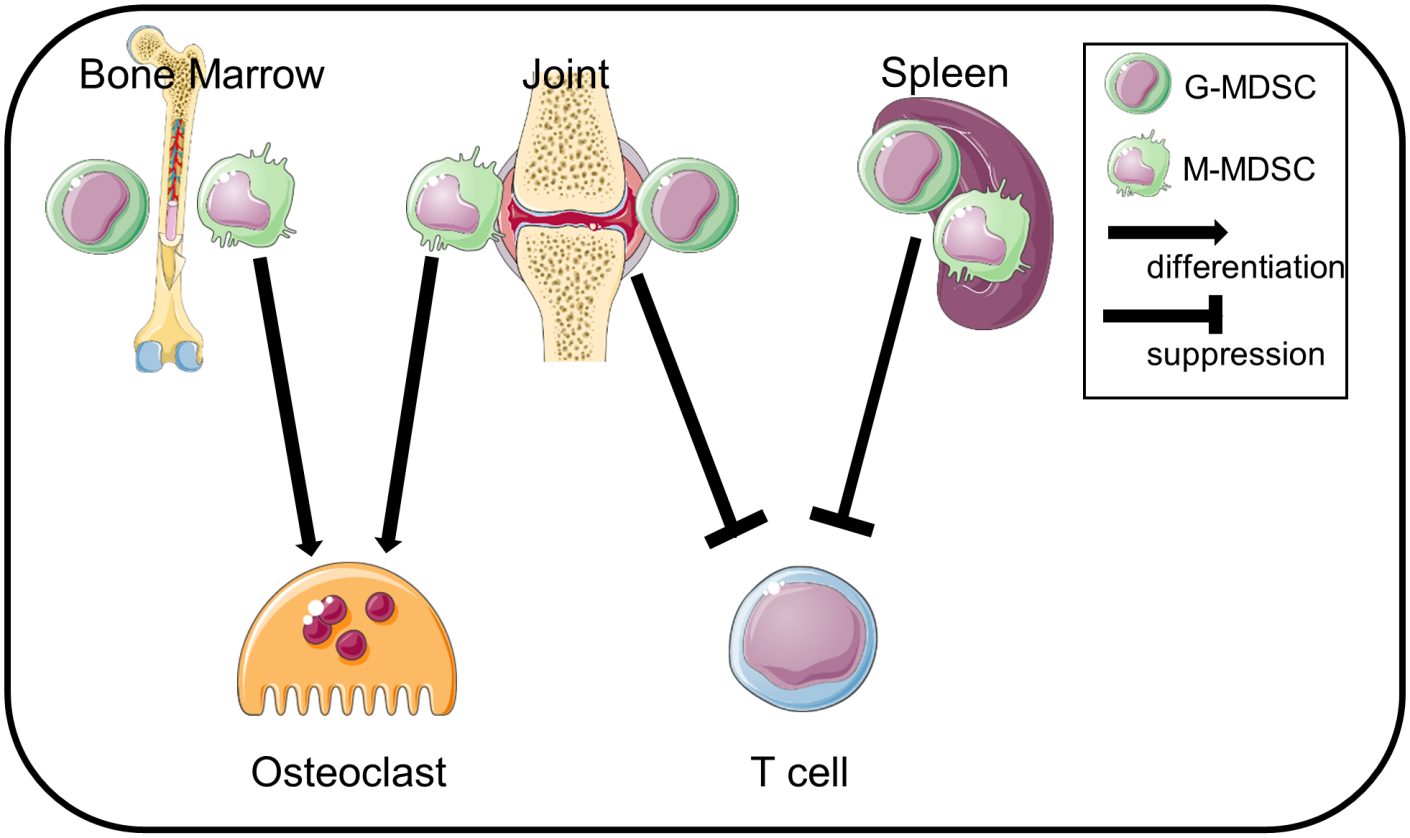
Schematic summary. Jo-MDSCs have dual functions: T cell suppression and osteoclast differentiation. BM-CD11b^+^Gr1^+^ cells differentiate into osteoclasts but are less suppressive. Sp-MDSCs suppress T cells but not differentiate into osteoclasts.

We summarize this study as a schematic in **Figure 6**. BM-CD11b^+^Gr1^+^ cells differentiate into osteoclasts but do not suppress T cell-proliferation, whereas Sp-MDSCs suppress T cell-proliferation but cannot differentiate to osteoclasts. In contrast, Jo-MDSCs have both attributes *in vitro* and bone resorptive function *in vivo*.

## 4 Discussion

We compared the CD11b^+^Gr1^+^ cells in the inflamed joints, in the BM or the spleen of SKG arthritic mice. The proportion of Jo-CD11b^+^Gr1^+^ cells was about 60% of total joint cells, most with an Ly6G^+^Ly6C^low^ (granulocytic) phenotype. In inflammatory diseases, different types of immune cells (e.g. neutrophils, macrophages, lymphocytes etc.) are found in inflamed organs (31). To confirm that CD11b^+^ Gr1^+^ cells were truly MDSCs, we stained them for several surface markers (CD11c, F4/80, MHC class II and CD80). In mice, MDSCs are phenotypically defined as CD11b^+^Gr1^+^CD11c^-^F4/80^int^CD124^+^ (6). Jo-/BM-/Sp-CD11b^+^Gr1^+^cells in this study did not express CD11c or F4/80. In addition, the lack of expression of MHC class II suggested that the CD11b^+^Gr1^+^ cells were not mature neutrophils, macrophages or dendritic cells. Low expression of CD80 on the CD11b^+^Gr1^+^ cells is consistent with the characteristics of MDSCs (22). We next morphologically assessed CD11b^+^Gr1^+^ cells by May-Grunwald-Giemsa staining, and identified two distinct cell types; cells with pseudo-segmented or ring-shaped nuclei (granulocytic CD11b^+^Gr1^+^ cells) and mononuclear cells (monocytic CD11b^+^Gr1^+^ cells). In mice, cells with ring-shaped nuclei have been reported to be immature MDSCs or precursors (32, 33). The granulocytic CD11b^+^Gr1^+^ cells did not have granules which is one of the characteristics of neutrophils. These phenotypical and morphological characteristics indicated that CD11b^+^Gr1^+^ cells in each organ of arthritic SKG mice were in agreement with a classification as MDSCs. Consistent with previous reports, these CD11b^+^Gr1^+^ cells from the different tissues were morphologically indistinguishable (34, 35).

By definition, MDSCs need to have suppressive function in addition to phenotypic and morphological characteristics (7). Hence, we investigated the suppressive function of CD11b^+^Gr1^+^ cells from each organ. Jo-CD11b^+^Gr1^+^ cells and Sp-CD11b^+^Gr1^+^ cells, but not BM-CD11b^+^Gr1^+^ cells, significantly suppressed T cell proliferation. These results indicated that Jo-/Sp-CD11b^+^Gr1^+^ cells were MDSCs and BM-CD11b^+^Gr1^+^ cells were IMCs. Few reports have focused on detailed functions of Jo-MDSCs in arthritic mouse models. It is known that MDSCs from peripheral blood and spleen in arthritic mice suppress T cell proliferation and Th17 differentiation (36) via mediators such as Arginase 1, iNOS and IL10 (10, 36). Our microarray analysis revealed that Jo-/Sp-MDSCs and BM-CD11b^+^Gr1^+^ cells strongly expressed *Cybb* (*Nox2*) and *Tgfb1*, but not *Nos2* and *IL10*. In addition, Jo-MDSCs expressed *CD274 (Pdl1)*, *Arg1*, *Egr2* and *Egr3* to a greater extent than BM-CD11b^+^Gr1^+^ cells or Sp-MDSCs, indicating that Jo-MDSCs also might use these molecules as suppressive factors. Egr2 and Egr3 are members of the zinc finger transcription factor family; their absence results in systemic autoimmunity in mice (37). Egr2 and Egr3 have been reported to suppress STAT1 and STAT3 activation by stimulating SOCS, and thus negatively regulate CD4^+^ T cells (38, 39). *Socs1* was upregulated in Jo-MDSCs in our microarray analysis (data not shown), which suggests that one of the suppressive mechanisms of Jo-MDSCs was passively via Egr2 and Egr3 (37). We assumed that the inflammatory environment in the joints could have contributed to the different characteristics of Jo-MDSCs relative to Sp-MDSCs or BM-CD11b^+^Gr1^+^ cells which are in lymphoid organs.

It is known that osteoclasts are differentiated from macrophage and DC precursor cells (MDPs), identified as CX3CR1^+^c-kit^+^Lin^-^Fllt3^+^cFms^+^, via some osteoclast precursors such as monocytes, macrophages or dendritic cells (40, 41). Furthermore, a recent study implied a role for MDSCs as osteoclast progenitors in cancer (42). That study showed that MDSCs from the BM of tumor-bearing mice with bone metastasis differentiated into osteoclasts *in vitro*, whereas MDSCs from LNs, blood, spleen or lung did not. Nitric oxide (NO) levels were elevated in MDSCs from the BM of tumor-bearing mice with bone metastasis, and an iNOS inhibitor suppressed the MDSCs to differentiate into osteoclasts. Consistent with this, our data showed that Jo-MDSCs tended to express more iNOS (*Tnfrsf11a*) than BM-MDSCs and Sp-MDSCs (**Supplementary Figure S3**), suggesting that Jo-MDSCs have a potential to differentiate into osteoclasts. Another study reported that MDSCs from BM of mice with collagen-induced arthritis differentiated into osteoclasts, but MDSCs from BM of non-arthritic controls did not. In that study, the canonical NF-κB pathway was critical for the BM-MDSCs to differentiate into osteoclasts (43). Our microarray data showed that Jo-MDSCs highly expressed DEGs related to the NF-κB pathway. Furthermore, we found that genes involved in the non-canonical NF-κB pathway such as *Nfkb2* and *RelB* were upregulated in Jo-MDSCs. Moreover, we demonstrated that Jo-MDSCs actually differentiated into osteoclasts *in vitro*. Then we would propose to name the Jo-MDSC derived-osteoclasts “Jo-MDSC-OCs”. Recently, it was reported that CX3CR1^high^Ly6C^int^F4/80^+^I-A/I-E macrophages, termed as arthritis-associated osteoclastogenic macrophages (AtoMs), can differentiate into osteoclasts in the inflamed synovium of arthritic mice (44). These cells have been proved as macrophages from the surface markers (F4/80^hi^) and its morphology (relatively large cells with foamy cytoplasm and cytoplasmic vacuoles). Although our Jo-CD11b^+^Gr1^+^ cells similarly differentiate into osteoclasts, they were considered to be distinct from AtoM due to their lack of expression of neither F4/80 or CX3CR1 (**Figures 1E, F**) and their completely different morphology (**Figure 1D**). Our study is, to the best of our knowledge, the first showing that Jo-MDSCs strongly expressing non-canonical NF-κB pathway-related genes and could differentiate into functional osteoclasts. These results may contribute to a better understanding of another mechanism behind bone destruction in RA inflammatory arthritis.

Our study has several limitations. First, we haven’t shown whether intra-articular injected Jo-MDSCs actually differentiated into osteoclasts and contributed to the bone resorption *in vivo*. In other words, we could not distinct Jo-MDSC-OCs from monocyte/macrophage derived OCs. Second, we don’t know the mechanism why Jo-MDSCs had both suppressive and osteoclastogenic functions *in vitro* but promoted inflammation and bone resorption *in vivo*. The local milieu of inflamed joints such as pro-inflammatory cytokines and RANKLs may affected the genetic or epigenetic changes in Jo-MDSCs because MDSCs have plasticity. Third, we have no data about whether Jo-MDSC-OCs exist in RA patients. We need to elucidate these questions in the future.

Despite these limitations, our study would contribute to understanding the role of MDSCs in inflammatory arthritis.

In summary, we identified MDSCs in inflamed joints of arthritic SKG mice. Jo-MDSCs expressed more CCR5 than BM-CD11b^+^Gr1^+^ cells and less CCR2 than Sp-MDSCs. Jo-MDSCs exhibited a different pattern of gene expression compared with BM-CD11b^+^Gr1^+^ cells or Sp-MDSCs. Jo-MDSCs highly expressed anti-inflammatory genes such as *CD274 (Pdl1), Arg1, Egr1 and Egr2* and suppressed T cell proliferation and differentiation *in vitro*. We also showed that Jo-MDSCs highly expressed NF-κB non-canonical pathway genes such as *Nfkb2* and *Relb* and could differentiate into functional osteoclasts *in vitro*. Finally, we found that intra articular injection of Jo-MDSCs promote joint destruction in arthritic SKG mice.

## 5 Conclusions

In this study, we demonstrated that MDSCs in inflamed joints have both suppressive function and osteoclast differentiation ability *in vitro*, and bone resorptive function *in vivo*.

## Data availability statement

The datasets presented in this study can be found in online repositories. The micro array data presented in the study are deposited in the DDBJ repository, accession number E-GEAD-685, https://ddbj.nig.ac.jp/public/ddbj_database/gea/experiment/E-GEAD-000/E-GEAD-685/.

## Ethics statement

The animal study was approved by the Institutional Animal Care and Use Committee of Kobe University (Permission number: P210508). The study was conducted in accordance with the local legislation and institutional requirements.

## Author contributions

YF, SS, APF, AM and JS contributed to conception and design of the study. HY, KU, YY and TN contributed the experiments. YF and SS performed statistical analysis. YF wrote the first draft of the manuscript. SS and JS reviewed and edited the draft. All authors contributed to the article and approved the submitted version.
