# Supplementary material for: Myeloid-derived suppressor cell-derived osteoclasts with bone resorption capacity in the joints of arthritic SKG mice
Source: Front Immunol. 2024 Mar 19;15:1168323. doi: 10.3389/fimmu.2024.1168323 (PMC10985135; doi:10.3389/fimmu.2024.1168323)

Supplementary Material

Myeloid-derived suppressor cell-derived osteoclasts with bone resorption capacity in the joints of arthritic SKG mice.

Yoshikazu Fujikawa, Sho Sendo

*** Correspondence:** Jun Saegusa: jsaegusa@med.kobe-u.ac.jp

1 Supplementary Figures and Tables

1.1 Supplementary Figures

Supplementary Figure 1. Representative images of flow cytometry of $CD11b^{+}Gr1^{+}$ cells in non-arthritic SKG mouse.

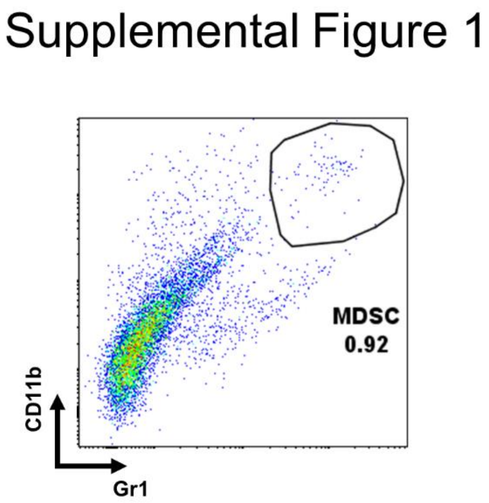

Supplementary Figure 2. Verification of the purity about sorted $CD11b^{+}Gr1^{+}$ cells from BM, spleen and joints.

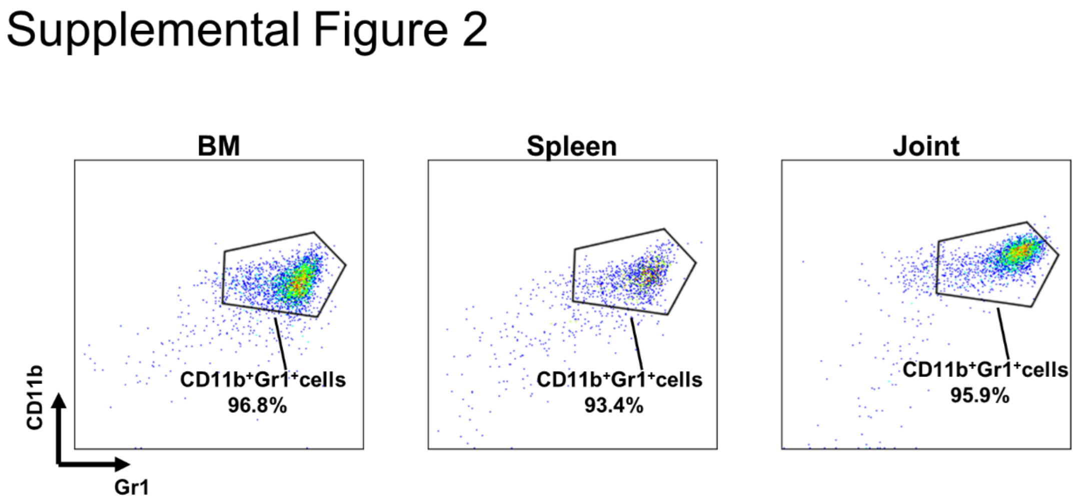

Supplementary Figure 3. A, mRNA expression levels of immunosuppressive genes (*Cybb* (*Nox2*), *Tgfb1*, *Nos2* (*iNOS*), and *Il10*) of isolated CD11b$^+$Gr1$^+$ cells from BM, spleen and joints, which were not differentially expressed among the three tissues. **B**, Scatter plot analysis of suppressive genes between BM- and Sp-CD11b$^+$Gr1$^+$ cells (left), and between Jo- and Sp-CD11b$^+$Gr1$^+$ cells (right).

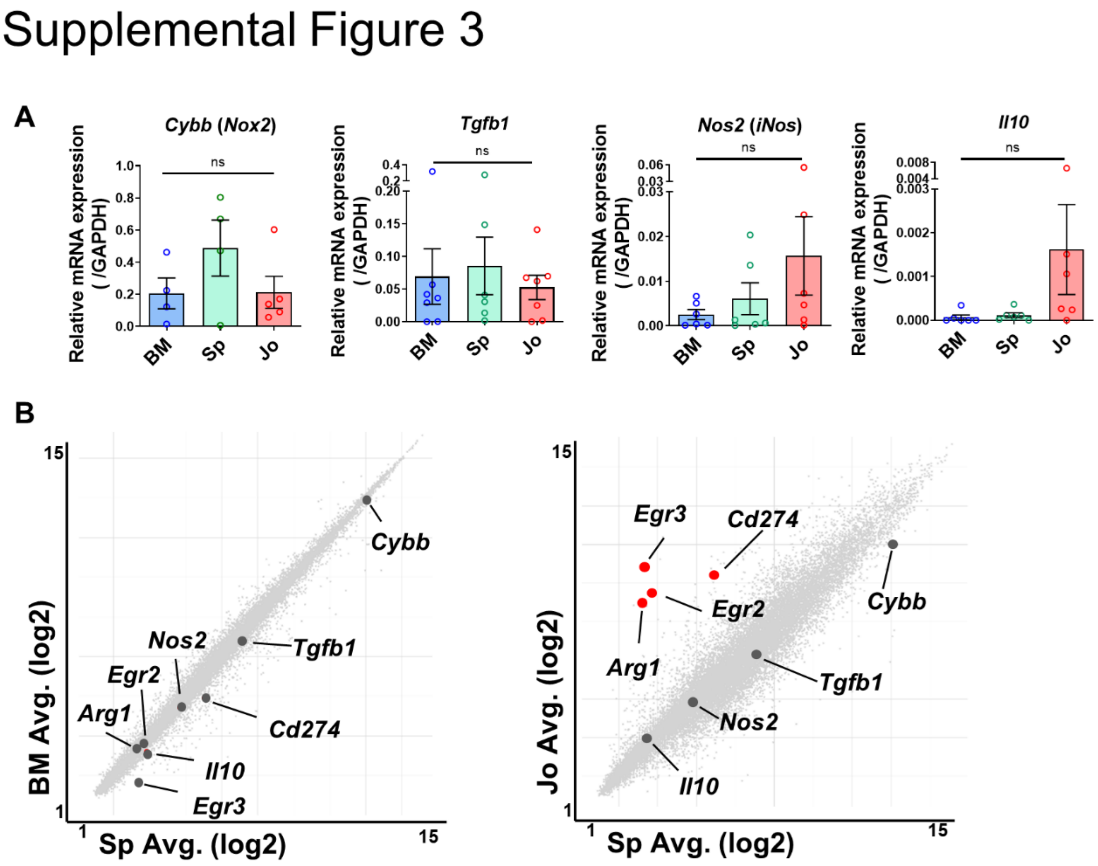

Supplementary Figure 4. Scatter plot analysis of genes relevant to NF-κB canonical pathway (*NFkb1*, *Rela*, *Traf2*, *Traf3* and *Map3k7*) between Jo- and Sp-CD11b$^+$Gr1$^+$ cells.

Supplemental Figure 4

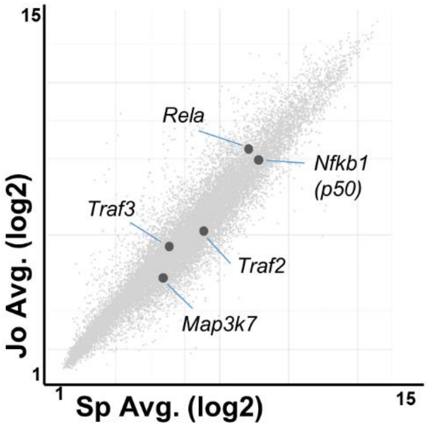

Supplementary Figure 5. A, mRNA expression levels of genes relevant to NF-κB noncanonical pathway (*Tnfrsf11a*, *Traf6* and *Map3k14*) of isolated CD11b$^+$Gr1$^+$ cells from BM, spleen and joints, which were not differentially expressed among the three tissues. **B**, Scatter plot analysis of genes relevant to NF-κB non-canonical pathway between Jo- and BM-CD11b$^+$Gr1$^+$ cells (left) or between BM- and Sp-CD11b$^+$Gr1$^+$ cells (right).

Supplemental Figure 5

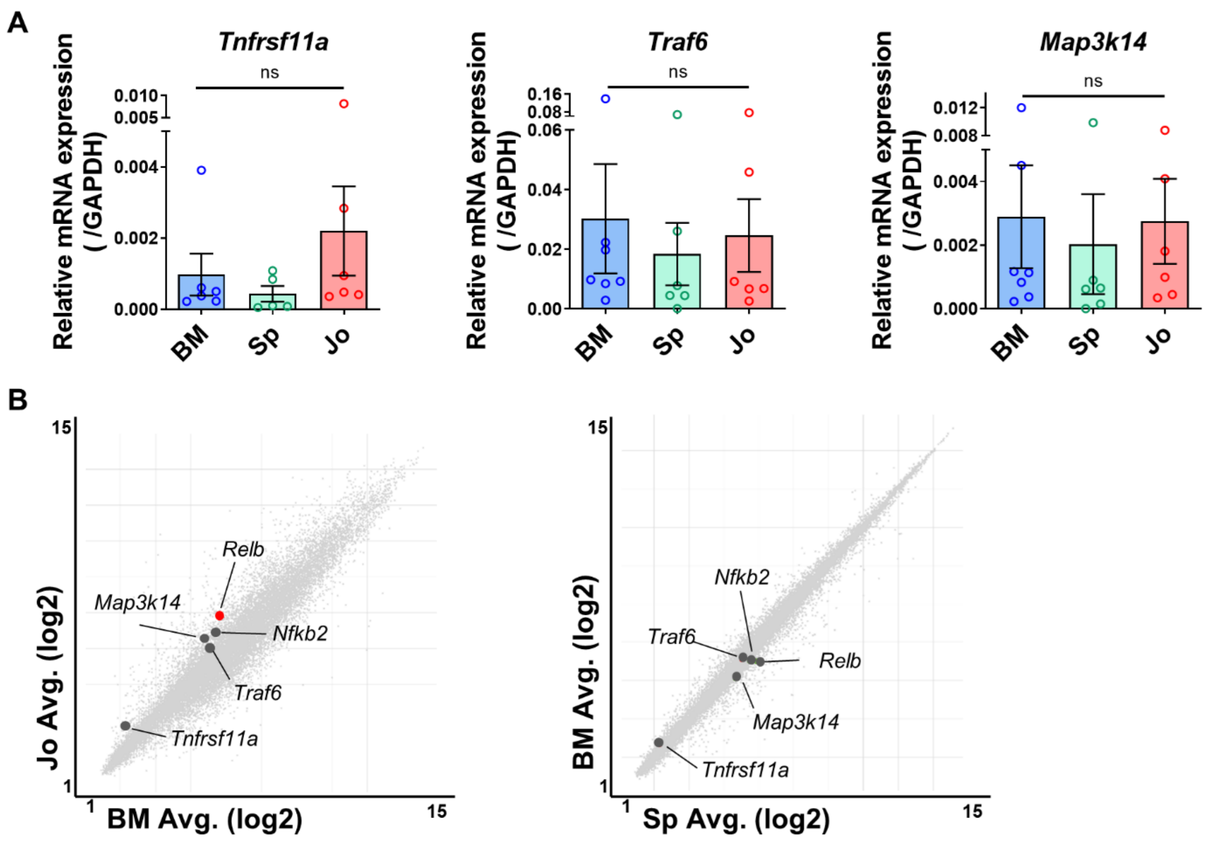

Supplement: Supplementary file 1 [file DataSheet_1.pdf]
